# Ramp lesions are frequently missed in ACL-deficient knees and should be repaired in case of instability

**DOI:** 10.1007/s00167-019-05521-3

**Published:** 2019-05-10

**Authors:** Alexander Bumberger, Ulrich Koller, Marcus Hofbauer, Thomas Manfred Tiefenboeck, Stefan Hajdu, Reinhard Windhager, Wenzel Waldstein

**Affiliations:** Department of Orthopaedics and Trauma Surgery, Vienna General Hospital, Medical University of Vienna, Waehringer Guertel 18-20, 1090 Vienna, Austria

**Keywords:** Ramp lesion, Meniscocapsular separation, Meniscocapsular attachment tear, ACL deficiency, Knee instability, Review

## Abstract

**Purpose:**

The aim of the current study was (1) to provide an overview of common definitions and classification systems of ramp lesions (RL) and (2) to systematically review the available literature with regard to the diagnosis and treatment of RLs in anterior cruciate ligament (ACL)-deficient knees.

**Methods:**

Following the PRISMA guidelines, MEDLINE and Scopus were searched for articles (1) reporting on acute or chronic ACL injuries, (2) with concomitant medial meniscus injury, (3) located at the posterior meniscocapsular attachment site (and red–red zone). Ex vivo studies, reviews and technical notes were excluded.

**Results:**

Twenty-seven studies were included based on the criteria mentioned above. RLs are common in ACL-deficient knees with a prevalence ranging from 9 to 24%. RLs should especially be suspected in younger patients, patients with an increased meniscal slope and in patients with prolonged time from injury to surgery. The sensitivity of MRI for the detection of RLs ranges from 48 to 86% at a specificity of 79–99%. For arthroscopy, RLs are easily missed through standard anterior portals (sensitivity 0–38%). RL repair leads to a significant improvement of subjective knee scores, regardless of the specific fixation technique. For stable RLs, the literature suggests equivalent postoperative stability for trephination and abrasion compared to surgical RL repair.

**Conclusion:**

Ramp lesions are frequently missed in ACL-deficient knees on standard arthroscopy with anterior portals only. If a RL is suspected, exploration via an additional posteromedial portal is indicated. In case of instability, RL repair should be performed.

**Level of evidence:**

IV.

## Introduction

The mechanisms of anterior cruciate ligament (ACL) rupture and concomitant knee injuries have been extensively investigated over the last decades, revealing that the minority of ACL ruptures occurs as an isolated injury [12, 20]. Meniscal tears have been reported to be present in 55% up to nearly 80% of ACL injuries, with significantly higher rates in chronically ACL-deficient knees [12, 26, 46]. Accordingly, time to surgery correlates with the incidence of concomitant meniscus injuries [25, 29]. In particular, the incidence of medial meniscus (MM) tears seems to significantly increase with delayed surgery [26, 50]. Conversely, patients with medial or lateral meniscus instabilities have a significantly increased risk of ACL failure after reconstruction [32]. Hence, early ACL reconstruction (ACLR) and meniscal repair are recommended to prevent secondary meniscus injuries and improve the long-term outcome [12, 27].

Over the last years, more attention has been paid to tears located at the posteromedial meniscocapsular junction. Even though first characterizations date back at least 35 years [13], these injuries frequently remained unnoticed in knees with ACL injuries. Since their first description, a range of terms has been used synonymously to describe these lesions, including meniscocapsular separation (MCS), meniscosynovial tear, hidden lesion (HL) and ramp lesion (RL). The term ramp lesion was first used by Strobel in 1988, defining it as “a special type of meniscal injury involving the peripheral attachment of the posterior horn of the medial meniscus, typically associated with an ACL deficiency”. Currently, there is no consensus regarding the definition of RLs [4] which has resulted in misleading descriptions. Some authors have also considered posteromedial meniscus tears in the red–red zone as ramp lesions [23, 43].

**Fig. 1:**
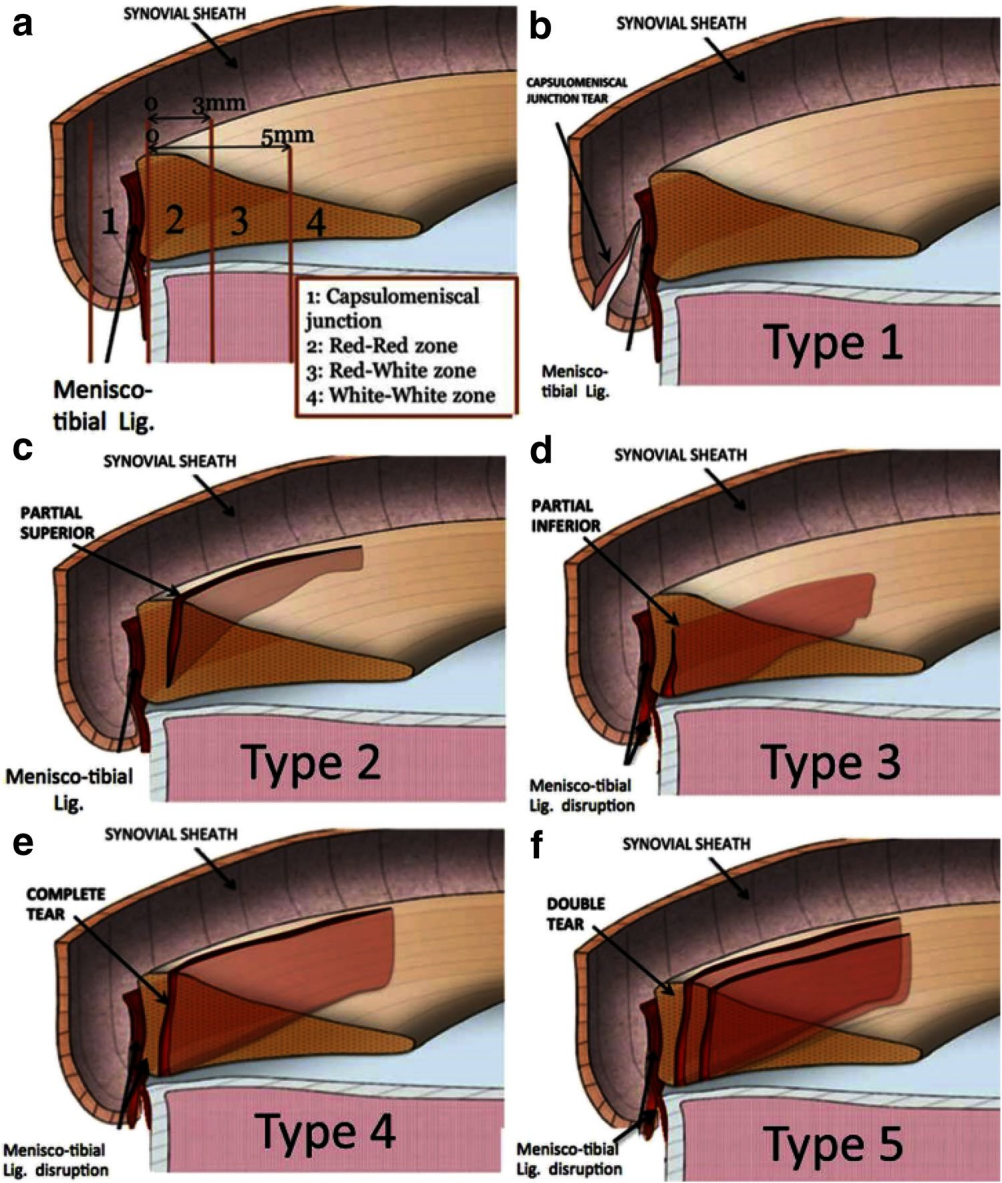
Illustration of five ramp lesion subtypes depending on tear pattern, location, degree of mobility and visibility during arthroscopy according to Thaunat et al. [43]

Thaunat et al. classified five lesion subtypes by their exact tear pattern, location, degree of mobility and visibility during arthroscopy [43]. Type 1 is meniscocapsular junction tears located in the synovial sheath with very low mobility at probing. Type 2 includes partial superior meniscus tears which are stable and can only be diagnosed via the trans-notch approach. Type 3 is partial inferior meniscus tears (hidden lesions) associated with meniscotibial ligament disruptions resulting in high probing mobility. Type 4 involves complete longitudinal vertical meniscus tears in the red–red zone. Type 5 describes double longitudinal vertical tears (Fig. 1).

An alternative classification has been proposed by Seil et al., paying more attention to the mediolateral extent of lesions and the properties of the capsule–ligament complex depending on the degree of flexion [34]. Furthermore, there seems to be confusion concerning the term “peripheral” in the context of RLs, since it has been referred both to the meniscocapsular and meniscotibial attachment sites of the PHMM [5, 9], as well as to lesions in the RR zone [23, 44]. Table 1 provides an overview of different RL definitions.

**Table 1:** Definitions of ramp lesions as described in the literature

Authors	Year	RL definition
Definitions not including RR zone tears		
Di Vico et al. [9]	2017	Ramp lesions and hidden lesions represent subgroups of longitudinal lesions involving the peripheral attachment of the PHMM
Seil et al. [34]	2017	Traumatic disruptions between the PHMM and its mensicoligamental junction
Chen et al. [5]	2017	Peripheral attachment lesion of the PHMM
Keyhani et al. [16]	2016	Longitudinal tear or detachment of the peripheral rim around the PHMM
Li et al. [22]	2015	Peripheral attachment lesion of the PHMM
Strobel et al. [42]	1988	Meniscal injury involving the peripheral attachment of the PHMM and is typically associated with an ACL deficiency
Definitions including RR zone tears		
Sonnery-Cottet et al. [40]	2018	Disruption or tear of the peripheral meniscocapsular attachments of the posterior horn of the medial meniscus (exclusion criteria suggests that RR zone is included)
Kim et al. [18]	2018	Peripheral longitudinal tear within 4 mm of the meniscocapsular junction of PHMM
Thaunat et al. [43, 44]	2016	Meniscosynovial or meniscocapsular tears + RR zone (see Fig. 1)
Furumatsu et al. [11]	2013	Peripheral longitudinal tears of the MM included partial- or full-thickness, simple longitudinal tears ≥ 1 cm in length located in the outer one-third of the posterior segment
Liu et al. [23, 24]	2011	Tear of the peripheral attachment of the posterior horn of the medial meniscus (synovial–meniscus junction or red–red zone)
Resnick et al. [31]	2007	Double vertical longitudinal tears of the PHMM

Some authors consider also lesions in the red–red zone as ramp lesion

**Fig. 2:**
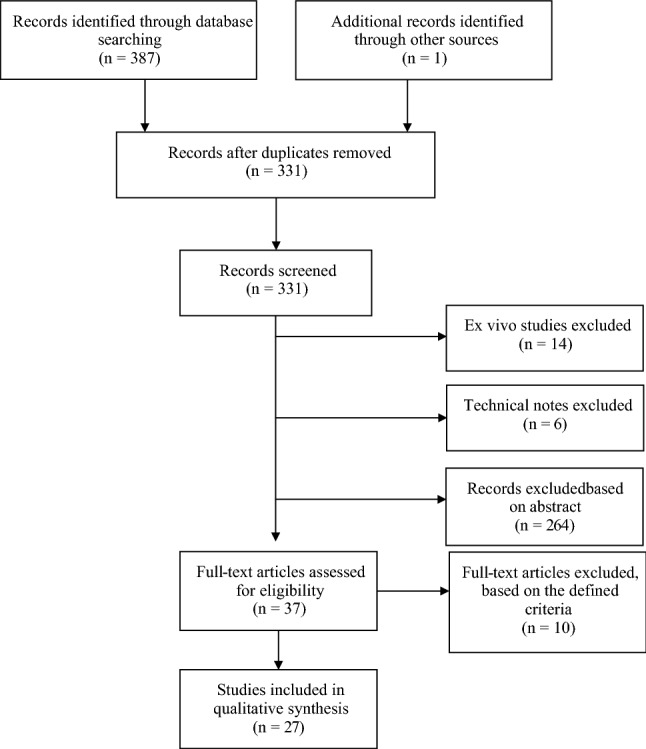
Flow chart of study protocol following the PRISMA guidelines. The systematic review included 27 studies

This review was conducted due to the growing number of publications regarding posteromedial meniscocapsular attachment tears, as well as the inconsistent nomenclature. The aim of this study was (1) to provide an overview of common ramp lesion definitions and classification systems and (2) to systematically review the available literature with regard to the diagnosis and treatment of ramp lesions (Fig. 2).

## Materials and methods

A systematic database search of the literature concerning ramp lesions in ACL-deficient knees utilising pre-defined search terms was performed (see addendum). The study was conducted according to the preferred reporting items for systematic reviews and meta-analysis (PRISMA) guidelines [28]. The methodological index for non-randomised studies (MINORS) was used to assess the quality of all included non-randomised studies. Included databases were Medline and Scopus. English and German articles were considered because the authors speak both languages.

The search terms (see addendum) were assembled to cover a broad spectrum of meniscus and associated knee injuries, in order not to miss any relevant literature. As represented in our search terms, we paid special attention to keywords such as “ramp”, “hidden”, “meniscocapsular”, “meniscosynovial” and “posteromedial” in the context of meniscus lesions. Next, relevant articles were preselected based on the abstract by two independent reviewers (AB and WW). A third reviewer (TT) was consulted to obtain consensus in case of discrepancy. Subsequently, the full text was carefully reviewed to check whether included lesions were (1) associated with acute or chronic ACL injury, (2) concerning the medial meniscus, and (3) located at the posterior meniscocapsular attachment site (and the periphery of the RR zone). Ex vivo studies, reviews and technical notes were excluded. Data regarding prevalence, diagnosis, surgical technique and outcome of RL repair were systematically extracted from the included studies.

## Results

A total of 387 studies matched our search terms. After exclusion of duplicates, 37 studies were initially included based on their abstract. 27 studies were finally included. The average MINORS score of all non-randomised studies was 8.7/16 for non-comparative studies and 16.9/24 for comparative studies, respectively (Tables 2, 3).

**Table 2:** Patient demographics and study details extracted from studies included in the review

References	Journal	Year	No. of patients	Gender (% male)	Mean age	Study type	Mean follow-up (months)	Focus	MINORS
Sonnery-Cottet et al. [40]	AMJSM	2018	3214	72.5	NR	Case–control	45.6	Epidemiology, Repair (SutureLasso)	17/24
Kim et al. [19]	AJR	2018	85	83.5	31.6	Retrospective analysis	4 days (trauma to MRI)	Diagnosis (MRI)	10/16
Seil et al. [35]	KSSTA	2018	224	64.3	27.0	Case–control	NR	Epidemiology	7/16
Edgar et al. [10]	JAAOS	2018	337	64.0^a^	23.0	Cross-sectional	NR	Epidemiology	7/16
Kumar et al. [21]	OJSM	2018	852	55.2	28.6	Case–control	NR	Epidemiology	16/24
Yeo et al. [48]	Skeletal Radiology	2018	78	82.1	33.7	Retrospective analysis	NR	Diagnosis (MRI)	16/24
Hatayama et al. [14]	Arthroscopy	2018	155	51.0	25.3	Case–control	42d (MRI to ASC)	Diagnosis (MRI)	18/24
Kim et al. [18]	Arthroscopy	2018	195	88.2	31.7	Case–control	9.3 (injury to surgery)	Diagnosis (ASC)	17/24
Di Vico et al. [9]	MLTJ	2017	115	90.4	27.0	Observational study	NR	Diagnosis (ASC)	11/16
Chen et al. [5]	JNS	2017	46	73.9	26.0	Observational study	32.0	Repair (FastFix, Smith&Nephew)	10/16
Yang et al. [47]	JMNI	2017	68	75.0	35.3	Case–control	18.0	Repair (Trephination/Abrasion vs FastFix, Smith&Nephew)	19/24
DePhillipo et al. [6]	AMJSM	2017	301	66.0^a^	29.6^a^	Case series	NR	Epidemiology, Diagnosis (MRI)	7/16
Arner et al. [2]	KSSTA	2017	90	50.0	28.0	Case series	57.3 days (MRI to ASC)	Diagnosis (MRI)	16/24
Malatray et al. [25]	KSSTA	2017	56	76.8	14.0	Case series	11.5 (trauma to surgery)	Diagnosis (ASC)	9/16
Liu et al. [24]	AMJSM	2017	91	74.7	35.2	RCT	24.0	Repair (Trephination/Abrasion vs Suture Hook, Linvatec)	
Keyhani et al. [16]	KSSTA	2017	128 (166)	83.6	24.0	Case series	24.0	Repair (Suture Hook, Linvatec)	13/16
Thaunat et al. [44]	Arthroscopy	2016	132	83.3	26.4	Case series	27.0	Repair (Suture Lasso, Arthrex)	12/16
Song et al. [38]	AMJSM	2016	106	81.1	26.1	Case–control	NR	Epidemiology (increased MM slope)	16/24
Zhang et al. [49]	Int. Orthopaedics	2015	78	60.3	26.7	Case series	NR	Wave sign chondral injury	6/16
Li et al. [22]	KSRR	2015	23	NR	NR	Case series	14.0	Repair (FastFix, Smith&Nephew)	6/16
Sonnery-Cottet et al. [39]	AMJSM	2014	302	78.8	28.0	Case series	9.7 (trauma to surgery)	Diagnosis (ASC)	8/16
Kijowski et al. [17]	Musculoskeletal Imaging	2014	64	62.5	25.2	Case series	22d (trauma to MRI), 48d (MRI to surgery), 70d (trauma to surgery)	Diagnosis (MRI)	6/16
Furumatsu et al. [11]	Int. Orthopaedics	2013	20	40.0	19.0	Case series	24.0	Repair (FastFix, Smith&Nephew)	12/16
Liu et al. [23]	AMJSM	2011	868	78.5^a^	24.7^a^	Cross-sectional	27.2 (trauma to surgery)	Epidemiology	7/16
Bollen et al. [3]	JBJS	2010	183	NR	NR	Cross-sectional	NR	Epidemiology	7/16
Smith et al. [37]	AJSM	2001	575	63.7	25.4	Cross-sectional	NR	Epidemiology	12/16
Rubin et al. [33]	Musculoskeletal Radiology	1996	52	75.0	32.0	Case series	46d (MRI to surgery)	Diagnosis (MRI)	6/16

*AMJSM* American Journal of Sports Medicine, *AJR* American Journal of Roentgenology, *JAAOS* Journal of the American Academy of Orthopedic Surgeons, *JBJS* Journal of Bone and Joint Surgery, *JMNI* Journal of Musculoskeletal and Neuronal Interactions, *JNS* The Journal of Knee Surgery, *JOSR* Journal of Orthopaedic Surgery and Research, *KSRR* Knee Surgery and Related Research, *KSSTA* Knee Surgery Sports Traumatology Arthroscopy, *MLTJ* Muscles, Ligaments and Tendons Journal, *OJSM* Orthopedic Journal of Sports Medicine, *OTSR* Orthopaedics and Traumatology: Surgery and Research

^a^Patients with RL

**Table 3:** Methodological index for non-randomised studies (MINORS)

References	1	2	3	4	5	6	7	8	9	10	11	12	Total
Sonnery-Cottet et al. [40]	2	2	2	2	0	2	1	0	2	2	0	2	17/24
Kim Y et al. [19]	2	2	1	2	0	1	2	0	–	–	–	–	10/16
Seil et al. [35]	2	1	2	2	0	0	0	0	–	–	–	–	7/16
Edgar et al. [10]	2	2	1	2	0	0	0	0	–	–	–	–	7/16
Kumar et al. [21]	2	2	1	2	2	0	0	0	2	2	1	2	16/24
Yeo et al. [48]	2	2	1	2	2	0	0	0	1	2	2	2	16/24
Hatayama et al. [14]	2	2	2	2	1	1	2	1	1	2	0	2	18/24
Kim et al. [18]	2	2	2	2	0	0	0	2	1	2	0	2	17/24
Di Vico et al. [9]	1	2	2	2	0	2	2	0	–	–	–	–	11/16
Chen et al. [5]	1	2	1	2	0	2	2	0	–	–	–	–	10/16
Yang et al. [47]	2	2	1	2	0	2	2	0	2	2	2	2	19/24
DePhillipo et al. [6]	2	2	1	2	0	0	0	0	–	–	–	–	7/16
Arner et al. [2]	2	2	0	2	2	0	0	0	2	2	2	2	16/24
Malatray et al. [25]	2	2	2	2	0	0	0	1	–	–	–	–	9/16
Keyhani et al. [16]	2	2	2	2	0	2	1	2	–	–	–	–	13/16
Thaunat et al. [44]	2	2	2	2	0	2	2	0	–	–	–	–	12/16
Song et al. [38]	2	2	0	2	2	0	0	0	2	2	2	2	16/24
Zhang et al. [49]	1	2	1	2	0	0	0	0	–	–	–	–	6/16
Li et al. [22]	0	0	0	2	0	2	2	0	–	–	–	–	6/16
Sonnery-Cottet et al. [39]	2	2	2	2	0	0	0	0	–	–	–	–	8/16
Kijowski et al. [17]	1	0	1	2	2	0	0	0	–	–	–	–	6/16
Furumatsu et al. [11]	2	2	0	2	2	2	2	0	–	–	–	–	12/16
Liu et al. [23]	2	2	1	2	0	0	0	0	–	–	–	–	7/16
Bollen et al. [3]	1	2	2	2	0	0	0	0	–	–	–	–	7/16
Smith et al. [37]	2	2	2	2	2	2	0	0	–	–	–	–	12/16
Rubin et al. [33]	2	0	1	2	1	0	0	0	–	–	–	–	6/16

2 = reported and adequate, 1 = reported, but inadequate, 0 = not reported; max. score 16 (non-comparative studies) and 24 (comparative studies)

### Epidemiology

The reviewed literature reports a prevalence of RLs in ACL-deficient knees ranging from 9 to 24% [3, 35]. RLs account for 17%–55% of all MM injuries [14, 26]. There is evidence that RLs occur more frequently in younger and paediatric patients [23, 25]. An increased medial meniscal slope [38] and prolonged time from injury to surgery [16, 23, 40] have also been associated with a higher incidence of RLs. Sonnery-Cottet et al. [40] reported that male sex, maximum age ≤ 30 years, revision ACL reconstruction (ACLR), side-to-side laxity difference > 6 mm, and the presence of a lateral meniscal tear are all significant risk factors for RLs. Furthermore, RLs seem to be more prevalent in contact sports injuries [35] (Table 4).

**Table 4:** Arthroscopic prevalence of ramp lesions in ACL-deficient knees

Authors	Year	*N*	Selection criteria	Mean age	TFI (months)	Tear type	Prevalence (%)
Sonnery-Cottet et al. [40]	2018	3214	Primary or revision ACLR	28.0	4.9	RL	23.9
Seil et al. [35]	2018	224	ACL injury	27.4	4.6	MM	41.0
						RL	55.0^a^, 24.0
Edgar et al. [10]	2018	337	Primary ACLR	26.0	NR	Posteromedial MCS	13.1
Yeo et al. [48]	2018	78	ACL injury	33.7	NR	RL	9.0
Kumar et al. [21]	2018	852	ACL injury	29.2	NR	MM	36.0
						RL	41.4^a^, 14.9
Kim SH et al. [18]	2018	195	Acute or chronic ACL injury	31.7	NR	RL	26.6
Mansori et al. [26]	2018	362	ACL injury	32.1	11.7	MM	40.6
						MM peripheral (MCS + RL)	5.0
Hatayama et al. [14]	2018	155	ACL injury	25.3	NR	MM	52.3
						RL	29.7^a^
Di Vico et al. [9]	2017	115	ACL injury	27.0	10.0	Longitudinal lesions that involve the peripheral attachment of the PHMM	9.6
						RL	7.8
						HL	1.7
DePhillippo et al. [6]	2017	301	ACL injury + MM tear	NR	NR	RL	16.6^a^
Malatray et al. [25]	2017	56	ACL injury	13.9	20.2	RL	23.2
Arner et al.	2017	90	ACLR	28.0	NR	RL	14.4
Song et al. [38]	2016	1012	Non-contact ACL injury	26.1 (RL only)	NR	RL	15.8
Keyhani et al. [16]	2016	927	ACL injury	24.0	NR	RL	17.9
Shelbourne et al. [36]	2015	3385	ACL injury	21.5	NR	Vertical tears in the periphery of the PHMM at least 1 cm in length	12.4
Peltier et al. [30]	2015	39	ACL injury	33.0	NR	Presumably RL + HL	12.8
Sonnery-Cottet et al. [39]	2014	302	ACL injury	NR	NR	MM	41.4
						RL	9.6
						HL	7.0
Liu et al. [23]	2011	868	ACL injury	NR	NR	RL	16.6
Bollen et al. [3]	2010	183	ACL injury	NR	NR	posteromedial MCS	9.3
Smith et al. [37]	2001	575	ACL injury + any meniscus tear	25.4	NR	Peripheral posterior horn tears of the medial meniscus	40^a^

*TFI* time from injury (to surgery); mean age and TFI are point estimates; percentages of prevalence refer to all included patients unless stated otherwise

^a^MM tears

### Arthroscopy

Most authors have pointed out that RLs are easily missed through a standard anterolateral arthroscopic portal [1, 3, 23, 39]. Thus, exploration via a trans-notch view or an additional posteromedial portal has been commonly suggested to specifically check for these lesions. The posteromedial portal has been reported to be safe concerning possible damage of popliteal neurovascular structures [5, 9, 16, 30].

Kim et al. evaluated the accuracy of a sequential arthroscopic 4-step approach for the diagnosis of RLs [18]. The authors showed that 38% of all RLs were found using the initial standard exploration via an anterolateral portal. Forty-eight percent of RLs were identified through an intercondylar view using a 30° arthroscope. A trans-notch view using a 70° arthroscope and exploration through a posteromedial portal both resulted in a 100% detection rate. Additionally, the authors did not find a significant correlation between prolonged time from injury (TFI) and the overall prevalence of RLs; however, an association between TFI and the diagnostic step of detecting a RL was observed. RLs in chronic ACL tears (> 3 months) were more often detected through a standard anterolateral portal as compared to those in acute ACL injuries (< 3 months).

The difficulty of diagnosing a RL via an anterolateral portal has been confirmed by others [9, 25, 39] (Table 5).

**Table 5:** Accuracy of different arthroscopic approaches and arthroscopes to diagnose ramp lesions

References	Journal	Year	No. of patients	Gender (% male)	Mean age	Study type	Mean follow-up (months)	Focus	Portal sensitivity	MINORS
Kim et al. [18]	Arthroscopy	2018	195	88.2	31.7	Case–control	9.3 (trauma to surgery)	Diagnosis (ASC/MRI)	AL (30°) 38%	17/24
									TN (30°) 48%	
									TN (70°) 100%	
									PM (NR) 100%	
Di Vico et al. [9]	MLTJ	2017	115	90.4	27.0	Observational	NR	Diagnosis (ASC)	AL (NR) 0%	11/16
									TN (NR) 82%	
									PM (NR) 100%	
Malatray et al. [25]	KSSTA	2017	56	76.8	14.0	Case series	11.5 (injury to surgery)	Diagnosis (ASC)	AL (30°) 8%	9/16
									TN (30°) 100%	
									PM (30°) 100%	
Zhang et al. [49]	Int. Orthopaedics	2015	78	60.3	26.7	Case series	NR	Wave sign chondral injury	NR	6/16
Sonnery-Cottet et al. [39]	AMJSM	2014	302	78.8	28.0	Case series	9.7 (trauma to surgery)	Diagnosis (ASC)	AL (30°) 0%	8/16
									TN (30°) 58%	
									PM (30°) 100%	

Lesions affecting the meniscotibial ligament can be covered by an intact capsule and, therefore, remain undetected unless soft-tissue debridement is performed [9]. In case of meniscus instability without a corresponding lesion on standard arthroscopy, debridement via a posteromedial portal under trans-notch visualisation is recommended [9]. Zhang et al. [49] reported an association between a wave-like chondral injury of the medial femoral condyle and the presence of RL on arthroscopic exploration. The authors examined 1596 patients of which 4.9% (78/1596) presented with the so-called wave sign. A RL was confirmed in all of these patients.

### Magnetic resonance imaging

Numbers regarding the sensitivity of MRI for the detection of RLs are very heterogeneous, ranging from 48 to 86% [6, 41]. The specificity has been reported to range from 79 to 99% [2, 48]. The criteria for the diagnosis of RLs on MRI in each individual study are displayed in Table 6. The diagnostic accuracy of hidden lesions on MRI remains unclear, since hidden lesions have not explicitly been considered in the aforementioned studies. Arthroscopy represented the gold standard for the diagnosis of RLs in all but one study; Kim et al. applied MRI as reference standard to demonstrate a correlation between the “uncovered medial meniscus sign” and an anterior tibial translation which could indicate instability of the PHMM in ACL-deficient knees [19]. The remainder of the studies applied similar methodologies, measuring the diagnostic accuracy of MRI in comparison to arthroscopy in an ACL-deficient patient collective (Table 6).

**Table 6:** Diagnostic accuracy of MRI in detecting ramp lesions relative to the arthroscopy via a posteromedial portal

References	Journal	Year	*N*	Gender (% male)	Mean age	Study type	Mean follow-up (months)	Focus	MRI sequence	Criteria for RL	Criteria on MRI	SEN/SPE/PPV/NPV	MINORS
Kim Y et al. [19]	AJR	2018	85	83.5	31.6	Retrospective analysis	4 days (trauma to MRI)	Diagnosis (MRI)	sag. T1w FSE, ax./sag./cor. T2w FS FSE, cor./ocor./sag. PDw 3D FSE, 3T	Peripheral meniscocapsular separation or a peripheral tear of the PHMM	Uncovered medial meniscus sign (anterior tibial translation)	SEN 84 SPE 95	10/16
Kumar et al. [21]	OJSM	2018	307	58.3	29.2	Case–control	27% < 8 weeks 60% > 8 weeks 13% unknown (trauma to surgery)	Diagnosis (MRI)	ax./cor./sag. T2 and PDw	Tears at the posterior medial meniscocapsular junction	PMTP edema (in at least 2 different planes)	PPV 55%.	16/24
Kim SH et al. [18]	Arthroscopy	2018	195	88.2	31.7	Case–control	9.3 (trauma to surgery)	Diagnosis (MRI/ASC)	NR	Peripheral longitudinal tear within 4 mm of the meniscocapsular junction of PHMM	NR	SEN 84% SPE 95% PPV 86% NPV 95%	17/24
Yeo et al. [48]	Skelet. Radiology	2018	78	82.1	33.7	Retrospective analysis	NR	Diagnosis (MRI)	sag. FS T2w, ax./cor. FS T2w or PDw, 3/1.5T	Tear in the attachment between the posteromedial meniscus and knee capsule	Irregularity at the posterior margin, complete fluid filling sign	SEN (irregularity at posterior margin) 86% SEN (complete fluid filling sign) 57% SPE (irregularity at posterior margin) 79% SPE (complete fluid filling sign) 92%	16/24
Hatayama et al. [14]	Arthroscopy	2018	155	51.0	25.3	Case–control	42 days (MRI to ASC)	Diagnosis (MRI)	cor. TSE FS PDw (TR/TE 2500/9.2), cor. TSE T1w (TR/TE 570/10), ax. gradient-echo T2* (TR/TE 680/16) 3 mm slice, sag.TSE FS PDw (TR/TE 3500/8.6) 2 mm slice; 3/1.5T	Longitudinal tears of the medial meniscus posterior horn (PHMM) around the meniscocapsular junction	High signal irregularity of the capsular margin or separation in the meniscocapsular junction of the PHMM on sagittal images	SEN 72% SPE 93%	18/24
DePhillipo et al. [6]	AMJSM	2017	301	66.0	29.6	Case series	5.7 weeks (trauma to surgery)	Epidemiology, diagnosis (MRI)	sag. PDw FS T2w, 3/1.5T	Tear of the peripheral attachment of the posterior horn of the medial meniscus at the meniscocapsular junction	NR	SEN 48%	7/16
Arner et al. [2]	KSSTA	2017	90	50.0	28.0	Case series	57.3 days (MRI to ASC)	Diagnosis (MRI)	sag. T2w, 3 mm slice; 1.5T	Posterior medial menisco- capsular separation	High signal irregularity with complete fluid filling between the posterior horn of the medial meniscus and capsular margin	SEN 46–85% SPE 92–99%	16/24
Kijowski et al. [17]	Musculoskelet. Imaging	2014	64	62.5	25.2	Case series	22 days (trauma to MRI), 48 days (MRI to surgery), 70 days (trauma to surgery)	Diagnosis (MRI)	ax. FS T2w FSE 3/1.5T (TR/TE, 3800/88 at 1.5T and 4050/85 at 3T), cor. interm.-w. FSE sequence (TR/TE, 1800/20 at 1.5 and 3T), cor. FS interm.-w. FSE (TR/TE, 2400/40 at 1.5 and 3T)	Peripheral vertical meniscal tear	NR	NR	6/16
Rubin et al. [33]	Musculoskelet. Radiology	1996	52	75.0	32.0	Case series	46 days (MRI to surgery)	Diagnosis (MRI)	sag. FSE, PDw (TR/TE 2500-3100/17-18, sag. T2w FSE (TR/TE 4000-5000/102-108), sag. dual-echo conventional spin echo (2300/15, 75); cor.FSE PDw (2000-2500/18-22), echo train length of four) and T2w (2800-4000/108); 1.5T	Detachment of the meniscal rim from the capsule	Meniscal edge displaced from the tibial margin or an area of signal intensity similar to that of fluid separated the meniscus from the capsule	PPV 8-10%	6/16

*FS* fat suppressed, *FSE* fast spin echo, *PDw* proton density weighted imaging, *T2w* T2 weighted imaging, *TSE* turbo spin echo, *ax.* axial, *sag.* sagittal, *cor.* coronal, *ocor.* oblique coronal

### Outcome

Eight studies were identified which report on the outcome of RL repair in a total of 855 ACL-deficient knees. The average age of patients at the time of surgery was 28.4 years and 74.9% (623/832) were males. Among the eight studies, one randomised controlled trial (RCT) [24], and two retrospective case–control studies were identified [40, 47] (Table 7).

**Table 7:** Overview of the clinical outcome following ramp lesion repair

Author	Year	Design	Study group	*N*	Selection criteria	Mean age	Technique	Mean follow-up (months)	Secondary meniscectomy rate
Sonnery-Cottet et al. [40]	2018	Case control	Observation	191	ACL + RL	28.0	ACLR + ALLR + RL repair	45.6	6.7%
			Control	225	ACL + RL	28.0	Isolated ACLR + RL repair	45.6	14.8%

									LNS pre-OP	LNS post-OP	LNS improvement
Chen et al. [5]	2017	Case series		46	ACL + RL	26.0	Smith&Nephew, Fast Fix	32 (14-36)	56.81 (37–70)	94.44 (90–99)	37.6
Yang et al. [47]	2017	Retrospective controlled	Observation	31	ACL + stable RL (max. 1-2 cm)	34.8	Abrasion and trephination only		64.2 ± 6.3	90.3 ± 8.7	26.1^a^
			Control	37	ACL + stable RL (max. 1-2 cm)	35.7	Smith&Nephew, Fast Fix	(At 12 and) 24 post-OP	66.2 ± 5.6	90.5 ± 5.8	24.3
Liu et al. [24]	2017	RCT	Observation	40	ACL + stable RL	35.6	All-inside (NR)	37.9 + − 15.9	68.6 ± 6.1	88.7 ± 4.8	20.1^a^
			Control	33	ACL + stable RL	34.8	Abrasion and trephination only	40.3 + − 16.5	64.3 ± 7.5	90.4 ± 5.8	26.1
Keyhani et al. [16]	2017	Case series		128	ACL + RL	24.0 (median)	ConMed-Linvatec, Lasso	Min. 24 (24-47)	61.7 ± 3.2	87.8 ± 3.9	26.1
Thaunat et al. [44]	2016	Case series		81	ACL + longitudinal PHMM tear (MCS or RR zone; or RR–RW junction)	28.2	Arthrex, SutureLasso	27 (24–29)	IKDC: 63.8 ± 13.5	IKDC: 85.7 ± 12	IKDC Improvement: 21.9
Li et al. [22]	2015	Case series		23	ACL + RL	NR	Smith&Nephew, Fast Fix	14 (6–27)	64.4 ± 4.52	91.2 ± 4.60	26.8
Furumatsu et al. [11]	2013	Case series		20	ACL + repair of MM peripheral longitudinal tears	19.0	Smith&Nephew, Fast Fix	24 (12–41)	60.1 ± 4.7	93.1 ± 3.1	33.0

*RCT* randomised controlled trial, *LNS* Lysholm Knee Score, *IKDC* International Knee Documentation Committee

^a^No significant difference as compared to control group

For RL repair, all studies used an all-inside technique. A variety of fixation systems (Smith & Nephew FasT-Fix [5, 22, 47], DePuy Synthes Omnispan [45], ConMed-Linvatec Suture-Hook and Arthrex Suture-Lasso [40, 44]) was used.

A range of different parameters regarding the outcome of RL repair in knees with concomitant ACL injuries has been reported in the literature. Among these are the Lysholm Knee Score (LNS), International Knee Documentary Committee (IKDC), Barrett criteria (presence of joint tenderness, effusion, and McMurray test) and healing status as per second-look arthroscopy or MRI. The LNS and IKDC Score were found to be the most frequently reported outcome parameters.

RL repair leads to a significant improvement of subjective knee scores, regardless of the specific technique (Table 7).

In a recent study by Sonnery-Cottet et al. [40] RL repair was performed in 769 patients suffering from acute or chronic ACL injury, using the Arthrex Suture Lasso. The patients were divided into the following two groups: ACLR only + RL repair; and ACLR + anterolateral ligament repair (ALLR) + RL repair. Failure of RL repair was defined as the performance of secondary meniscectomy within the follow-up period of an average 45.6 months. The authors reported that patients who underwent ACLR + ALLR had a > twofold lower risk for reoperation due to failure of RL repair as compared to patients who underwent ACLR + RL repair. This study demonstrates that ALLR can have a protective effect on RL repair; yet, the effect of RL repair itself was not analysed.

Liu et al. conducted a randomised controlled trial, including 73 patients with ACL injury and a concomitant stable RL [24]. Criteria for stable RLs included a lack of excessive anterior translation of the PHMM on probing from the anteromedial portal and a maximum lesion length of 1.5 cm measured from the posteromedial portal. Patients underwent all-inside surgical repair (Suture hook, Linvatec) or abrasion and trephination only. Patients were followed up for at least 24 months evaluating both subjective and objective parameters. In terms of functional scores (LNS and IKDC), there was no significant difference between the two groups. Additionally, no difference in knee stability (pivot-shift test, Lachmann test, KT-1000 arthrometer) was observed. Finally, healing status was assessed on T2-weighted sagittal MRI scans, showing that there was no statistically significant difference between the study group and control group. In conclusion, this study indicates that all-inside repair of stable RL is non-superior to abrasion and trephination alone.

In a retrospective controlled analysis, Yang et al. compared arthroscopic abrasion and trephination and Fast-Fix repair of RLs measuring 1–2 cm with concomitant ACL injury in 68 patients [47]. At a minimum of 24 months after surgery, there was no significant difference between the groups, indicating that arthroscopic refreshment of stable RLs achieves similar results compared to Fast-Fix repair (Table 7).

## Discussion

The most important finding of this study was that ramp lesions are frequently missed in ACL-deficient knees and should be repaired in case of instability.

Considering the high prevalence of RLs in ACL-deficient knees, exploration of the posteromedial meniscocapsular complex through a posteromedial (PM) portal seems indicated if a lesion is suspected [1, 15, 24, 40]. Recently, Kim et al. demonstrated that a trans-notch view using a 70° arthroscope can achieve similar results in terms of RL detection rates [18]. However, an additional posteromedial portal should be considered the gold standard for the diagnosis of ramp lesions. Furthermore, a PM portal offers the benefit of a full exploration of the lesion extent and allows for a dynamic assessment of instability in flexion. If meniscus instability is observed at probing and no obvious lesion can be identified, soft-tissue debridement via a posteromedial portal under trans-notch visualisation could aid the identification of hidden lesions [9].

The current literature depicts limited and quite heterogeneous data on the diagnostic accuracy of MRI to detect RLs. Arner et al. demonstrated that MRI can be useful to rule out RLs [2]. MRI has a lower sensitivity and accuracy for RLs than for meniscus body tears [14]. On MRI, fluid filling between the posterior horn of the medial meniscus and the capsular margin can be indicative of a RL [14, 48].

Based on the available case series, RL repair leads to a significant improvement of subjective knee scores, regardless of the specific technique. With LNS improvements between 20.1 [24] and 37.6 [5], most authors report significantly higher scores postoperatively as compared to preoperatively. These findings are in accordance with the results of a few case series reporting excellent healing results from 87 to 95% as evaluated during second-look arthroscopy [5, 24]. Biomechanical studies have also demonstrated that RL repair can restore knee stability when simultaneous ACLR is performed [7, 41]. A recent study by Sonnery-Cottet et al. demonstrated a significantly decreased risk of RL repair failure in patients undergoing concomitant ACLR and anterolateral ligament reconstruction, as compared to concomitant ACLR only [40]. For stable RLs, repair tends to be non-superior to trephination and abrasion only [24, 47]. This has also been shown by the only available randomised controlled study by Liu et al. [24].

There is a considerable inconsistency regarding the nomenclature of ramp lesions in the literature [34, 43]. The term ramp lesion has frequently been used as an umbrella term indicating posteromedial meniscus tears, rather than a specific type of tear (Table 1). Both tears of the meniscocapsular and meniscotibial attachment sites of the PHMM, as well as tears in the RR zone, have been termed ramp lesions [5, 9, 23, 43]. To allow a comparison of studies, the authors of this work suggest to only consider meniscocapsular and meniscotibial lesions as ramp lesion. This is supported by a recent experimental study of DePhilippo et al. reporting that the meniscocapsular and meniscotibial attachments merge at a common attachment at the PHMM [8].

The following limitations have to be acknowledged: (1) generalised recommendations for the treatment of ramp lesions cannot be made based on the available literature. Ramp lesion repair leads to significant improvements in the clinical outcome; however, only two studies compared different surgical treatment options (trephination and abrasion vs RL repair). (2) Different ACLR techniques might lead to a different outcome. This review did not account for different fixation techniques. (3) Some authors considered lesions of the meniscocapsular attachment site and lesions at the periphery of the red–red zone as ramp lesions. Therefore, in a limited number of cases (Table 1), the reported postoperative improvements of RL repair may not exclusively be related to meniscocapsular lesions. (4) None of the MRI studies considered hidden lesions explicitly; therefore, it remains unclear whether these lesions can preoperatively be detected on MRI.

This work reports on the high prevalence of peripheral posteromedial meniscus lesions in ACL-deficient knees and provides an overview of common ramp lesion classification systems. A better understanding of the preoperative and intraoperative diagnostic accuracy of ramp lesions is of high clinical relevance. In daily practice, a thorough investigation of the posteromedial meniscus should always be performed during ACL reconstruction. Repair is indicated in case of instability.

## Conclusion

Ramp lesions are common in ACL-deficient knees and are often missed through standard anterior portals. Exploration of the meniscocapsular complex via an additional posteromedial portal is recommended, if MRI suggests a lesion in this area or if instability is present at probing. If the posteromedial view still does not reveal any lesion despite significant instability, soft-tissue debridement might uncover hidden lesions (meniscotibial ligament disruptions).

Generalised recommendations for the treatment of ramp lesions cannot be made based on the available literature. Ramp lesion repair leads to a significant improvement of subjective knee scores, regardless of the specific fixation technique. For stable ramp lesions, trephination and abrasion might be equivalent to ramp lesion repair in terms of postoperative stability. In case of instability at probing, repair should be performed.

## Appendix

### Database query strings

#### PubMed

(tibial meniscus injuries[MeSH Terms] AND (“ramp” OR “hidden” OR “meniscocapsular” OR “meniscosynovial” OR “posteromedial”)) OR

(“ramp”[Tiab] AND lesion[Tiab] AND (meniscus[Tiab] OR meniscal[Tiab])) OR

(hidden[Tiab] AND lesion[Tiab] AND (meniscus[Tiab] OR meniscal[Tiab])) OR

(meniscocapsular[Tiab] AND (lesion[Tiab] OR “tears”[Tiab] OR “tear”[Tiab] OR “separation”[Tiab])) OR

(meniscosynovial[Tiab] AND (lesion[Tiab] OR “tears”[Tiab] OR “tear”[Tiab] OR “separation”[Tiab])) OR

(peripheral [Tiab] AND meniscus[Tiab] AND (“medial”[Tiab] OR “posteromedial”[Tiab]) AND (“tears”[Tiab] OR “tear”[Tiab] OR “separation”[Tiab]))

AND (English[lang] OR German[lang])

#### Scopus

TITLE-ABS-KEY ( ( ramp AND lesion ) OR ( meniscocapsular AND ( separation OR tear OR lesion OR injury ) ) OR ( meniscosynovial AND ( separation OR tear OR lesion OR injury ) ) OR ( hidden AND lesion ) OR meniscotibial AND ligament AND ( separation OR tear OR lesion OR injury ) )

## Abbreviations

ACL: Anterior cruciate ligament
ACLR: Anterior cruciate ligament reconstruction
ALLR: Anterolateral ligament repair
HL: Hidden lesion
IKDC: International Knee Documentation Committee
LNS: Lysholm Knee Score
TFI: Time from injury
RL: Ramp lesion
MCS: Menisco-capsular separation
PHMM: Posterior horn of the medial meniscus
